# Neuroanatomical Correlates of Intelligence in Healthy Young Adults: The Role of Basal Ganglia Volume

**DOI:** 10.1371/journal.pone.0093623

**Published:** 2014-04-03

**Authors:** Cosima Rhein, Christiane Mühle, Tanja Richter-Schmidinger, Panagiotis Alexopoulos, Arnd Doerfler, Johannes Kornhuber

**Affiliations:** 1 Department of Psychiatry and Psychotherapy, University Hospital, Friedrich-Alexander-University Erlangen-Nuremberg, Erlangen, Germany; 2 Department of Psychiatry and Psychotherapy, Klinikum rechts der Isar der Technischen Universität München, Munich, Germany; 3 Department of Neuroradiology, University Hospital, Friedrich-Alexander-University Erlangen-Nuremberg, Erlangen, Germany; Beijing Normal University, Beijing, China

## Abstract

**Background:**

In neuropsychiatric diseases with basal ganglia involvement, higher cognitive functions are often impaired. In this exploratory study, we examined healthy young adults to gain detailed insight into the relationship between basal ganglia volume and cognitive abilities under non-pathological conditions.

**Methodology/Principal Findings:**

We investigated 137 healthy adults that were between the ages of 21 and 35 years with similar educational backgrounds. Magnetic resonance imaging (MRI) was performed, and volumes of basal ganglia nuclei in both hemispheres were calculated using FreeSurfer software. The cognitive assessment consisted of verbal, numeric and figural aspects of intelligence for either the fluid or the crystallised intelligence factor using the intelligence test *Intelligenz-Struktur-Test* (I-S-T 2000 R). Our data revealed significant correlations of the caudate nucleus and pallidum volumes with figural and numeric aspects of intelligence, but not with verbal intelligence. Interestingly, figural intelligence associations were dependent on sex and intelligence factor; in females, the pallidum volumes were correlated with crystallised figural intelligence (r = 0.372, p = 0.01), whereas in males, the caudate volumes were correlated with fluid figural intelligence (r = 0.507, p = 0.01). Numeric intelligence was correlated with right-lateralised caudate nucleus volumes for both females and males, but only for crystallised intelligence (r = 0.306, p = 0.04 and r = 0.459, p = 0.04, respectively). The associations were not mediated by prefrontal cortical subfield volumes when controlling with partial correlation analyses.

**Conclusions/Significance:**

The findings of our exploratory analysis indicate that figural and numeric intelligence aspects, but not verbal aspects, are strongly associated with basal ganglia volumes. Unlike numeric intelligence, the type of figural intelligence appears to be related to distinct basal ganglia nuclei in a sex-specific manner. Subcortical brain structures thus may contribute substantially to cognitive performance.

## Introduction

Brain volume as an important factor for intelligence has always been a highly discussed topic. Higher levels of intelligence are thought to be associated with greater brain size [1] and increased total [2] and frontal grey matter volumes [3]. Recent studies suggest that neural correlates of intelligence may be distributed throughout the brain and consider the intelligence construct in a differentiated way [4], [5]. The prefrontal cortex, which is regarded as the predominant region of cognition, exhibits a strong connection with subcortical brain regions, specifically the basal ganglia, which were considered to be mainly involved in motor processes. This fronto-striatal circuit affects important executive functions of the brain [6]. In rodents, it has been shown that the striatum is involved in the acquisition and consolidation of goal-directed actions [7]. But also an independent role of subcortical structures for higher cognitive functions has recently become evident. Structural and functional studies as well as analyses of connecting pathways demonstrate an association between the basal ganglia and intelligence. The morphology of the right striatum was found to be correlated with fluid and spatial intelligence in healthy young adults [8]. Working memory related to fluid intelligence was shown to be influenced by the activity of the basal ganglia, that are supposed to function as a gate keeper to store only relevant information [9]. Analyses of white matter microstructures revealed a strong association of enhanced anatomical connectivity between frontal lobes and basal ganglia with intelligence performance, especially math-giftedness, fluid and spatial aspects [10]. Evidence for an important role of the basal ganglia in cognitive performance was also obtained from studies on cancer and neuropsychiatric diseases. Tumors in the basal ganglia region seem to influence intelligence performance. Patients with basal ganglia tumors reached significantly poorer results regarding general intelligence compared to those suffering from tumors in the pineal or suprasellar regions [11]. An impaired basal ganglia system underlying disease pathophysiology, such as in Parkinson’s disease and Huntington’s disease, also results in decreases in executive functions [12]–[14] and visual learning tasks [15]. In autism, the volume of the caudate nucleus is increased, which may indicate its involvement in an abnormally distributed network or provide insight into the origin of distinct autistic behaviours [16].

Taken together, it was only recently that the basal ganglia were regarded as important brain structures for distinct intellectual aspects, indicating the complexity and unexpected construction of the brain. Still missing were analyses that clarify the relationship between the volumes of the basal ganglia nuclei and specific aspects of intelligence in a non-clinical sample, which is aimed by the present study. To cope with the complexity of the intelligence construct, different aspects of intelligence were assessed to obtain a more detailed picture. The intelligence test employed enables the segregation of cognitive abilities into fluid and crystallised intelligence factors [17], where each of these factors is further divided into verbal, numeric and figural aspects [18]. This approach results in a detailed map of cognitive abilities that address both the processing speed, which is related to problem-solving and abstract logic (‘fluid intelligence’) for verbal, numeric and figural issues, as well as accumulated knowledge and experience (‘crystallised intelligence’) for all three aspects. Volumetric measurements of the striatum and pallidum were obtained using magnetic resonance imaging (MRI) brain scans. In this study, young healthy individuals were investigated. Further studies could extend these analyses to groups of patients to derive diagnostic and therapeutic aspects from the correlation between intelligence and basal ganglia volumes.

This study on healthy young adults provides evidence for a strong association between basal ganglia volumes and figural intelligence independent of the prefrontal cortex; however, this relation was highly influenced by sex. We also found a significant positive correlation between numeric crystallised intelligence and the right caudate nucleus in both females and males. Thus, subcortical brain structures are supposed to be required for spatial-numeric intelligence tasks. The sex-specific effect indicates that females and males may encode spatial information in a different manner.

## Experimental Methods

### Ethics Statement

The study was approved by the Ethics Committee of the Friedrich-Alexander-University of Erlangen-Nuremberg and conducted in concordance with the Declaration of Helsinki. Written informed consent was obtained from all participants.

### Subjects

In the GENES study at the University of Erlangen-Nuremberg, 156 healthy subjects were enrolled [19]–[21]. For 137 subjects of these participants both an intelligence assessment and MRI were available. Exclusion criteria included current or past medical, neurological or psychiatric conditions, history of head trauma with a loss in consciousness, substance abuse, positive family history of a neurological or psychiatric disease in first degree relatives, birth complications, any contraindications of having an MRI, history of heart or brain surgery, pregnancy or lactation. Ninety-one females (66%) and 46 males (34%) with a mean age of 24.5 years (range 19–35) and a mean of 12.8 years of education (range 9–17) were considered for this study.

### Structural Neuroimaging and Image Analysis

Three-dimensional structural MRI scans were acquired on a 1.5-Tesla scanner (Siemens Sonata, Erlangen, Germany). Axial FLAIR images with a slice thickness of 5 mm were obtained to exclude any intracranial pathology and reviewed by the neuroradiology department at the University of Erlangen-Nuremberg. Volumetric sagittal T1-weighted magnetisation prepared gradient echo (MPRAGE) images were obtained using the following parameters: repetition time (TR) = 2030 ms, echo time (TE) = 3.93 ms, slice thickness (SL) = 1 mm, field of view (FOV) = 290×290 mm, slices = 144, slice thickness = 1.0 mm, pixel size = 1×1 mm^2^ (voxel size = 1×1×1 mm^3^), and matrix size = 256×256. The T1-weighted images were converted from DICOM to the NIfTI format. Images were processed and subcortically segmented using FreeSurfer 5.1.0 software [22]. The procedure automatically includes intensity and motion correction, skull stripping, Talairach-transformation, intensity normalisation, subcortical processing and volumetric segmentation. Volumes of each subcortical structure for the left and right hemisphere were extracted and normalised against the intracranial volumes (ICV), which were derived from the sum of the grey matter, white matter and ventricle volumes to eliminate individual brain size differences [23], [24].

To ensure the quality of FreeSurfer results, hippocampal volumes calculated with FreeSurfer were compared with those obtained by tracing manually on a slice-by-slice basis by two independent and blinded operators [19], [20]. The correlation between both ways of assessing the hippocampal volumes was highly significant (left: r = 0.702, p<0.001; n = 145; right: r = 0.797, p<0.001, n = 145). FreeSurfer segmentation results from 28 randomly chosen subjects (20%) were visually inspected by two independent researchers blinded with respect to the individuals' data. No errors were noted and thus no further editing was performed. In addition, comparison of data extracted from two independent scans of the same three individuals correlated with r = 0.995. Moreover, data from repeated analysis of three datasets with FreeSurfer based on random seeds as starting values showed a high correlation of r = 0.999.

### Cognitive Performance Assessment

The full version of the *Intelligenz-Struktur-Test* I-S-T 2000 R [18] was used for cognitive assessment. The I-S-T 2000 R allows the assessment of crystallised and fluid intelligence and further subdivides these intelligence factors into verbal, numerical and figural aspects of intelligence performance. Psychological testing was performed in a standardised setting for the number of participants (n = 10 each), time of the day, duration of the assessment (40 min and 90 min for the crystallised and fluid intelligence tests, respectively) and test room; the psychometric testing was performed by an experienced psychologist (CR). The raw intelligence scores were normalised for age and education and transformed into standard values (*Standardwerte* SW; population mean = 100, SD = 10).

### Statistical Analysis

For statistical analysis the SPSS software (Statistical Package for the Social Sciences, Chicago, IL, for Windows, Version 21.0) was used. Due to reported sex differences in brain volumes and specific cognitive skills [25], analyses were conducted separately for females and males. The significance of differences regarding the demographic data was calculated using the t-test and chi-square test. Deviations from the normal distribution of the data were tested using the Kolmogorov-Smirnov test. Correlations between the intelligence scores and volumetric parameters were calculated using Pearson’s correlation coefficient (bivariate and partial). Correlations were considered as significant with p-values ≤0.05 (two-tailed). To correct for multiple testing we applied the widely-used concept of the false discovery rate (FDR) [26] with a moderate q-value of 0.2, which is a sensitive statistical threshold for determining changes in exploratory analyses [27]. To determine significant differences between two correlation coefficients we applied Fisher’s r-to-z transformation and used the chi-square test with a significance level of p≤0.05 (two-tailed).

## Results

### Demographic Results

Our study consisted of 137 healthy young individuals who were randomly divided into two test groups. One group (n = 67) was tested for fluid intelligence scores, and the other group (n = 70) was examined for crystallised intelligence scores. The number of individuals, number of females and males, age and education did not significantly differ between the two groups (Table 1). The homogeneity of the two groups allowed for an unbiased analysis of the volumetric and intelligence measurements in our sample.

**Table 1:** Demographic data.

	Female		Male	
	Fluid testing	Crystallised testing	Fluid testing	Crystallised testing
**N**	42	49	25	21
**% total N**	30.7	35.8	18.2	15.3
**Age**	24.2±3.3	24.0±2.9	25.8±3.6	25.0±3.3
**Years at school**	12.8±1.0	12.7±0.9	12.9±0.9	12.9±0.4

Values indicate the mean ± standard deviation. No significant differences between the test groups exist.

### The Correlation between Figural Intelligence and Basal Ganglia Volume is Influenced by Sex and Intelligence Factor

Measurements of our exploratory analysis revealed a nominally significant association between the volumes of the caudate nucleus, putamen and pallidum and figural intelligence scores. Interestingly, the strength of correlation between these nuclei and figural intelligence was highly affected by sex. Females showed significant correlations of the putamen and pallidum volumes and figural intelligence scores when crystallised aspects were considered. These effects were significant for the volumes of the total, left and right putamen and pallidum, respectively (Table 2). For the fluid figural measurements, no significant correlations were detected in females, with the exception of the left pallidum. In contrast, in males, significant correlations were observed for the volumes of the total, left and right caudate nucleus, and only for the fluid figural intelligence measurements (Table 2). For the crystallised figural intelligence scores, no significant associations were found in males. Significant correlations (p≤0.05; uncorrected) retained significance after FDR correction at q = 0.2 (critical p = 0.08).

**Table 2:** Correlations between the basal ganglia nuclei volumes and different aspects of intelligence.

		Figural intelligence				Numeric intelligence				Verbal intelligence			
		female		male		female		Male		female		male	
		fluid	Crystallised	fluid	crystallised	fluid	crystallised	fluid	crystallised	fluid	crystallised	fluid	crystallised
**Region**													
**Caudate**	**total**	−.141	.139	**.507** **	.295	.021	**.288** *	.170	**.430** *	−.025	.170	.227	.187
	**left**	−.081	.094	**.546** **	.341	.059	.247	.246	.389	−.027	.147	.248	.193
	**right**	−.192	.171	**.453** *	.245	−.016	**.306** *	.090	**.459** *	−.023	.180	.199	.177
**Putamen**	**total**	.076	**.318** *	.263	−.083	.042	.202	.071	.299	.098	.092	.123	.011
	**left**	.123	**.336** *	.187	−.102	.096	.206	−.082	.280	.147	.088	.005	−.028
	**right**	.018	**.287** *	.325	−.061	−.024	.190	.227	.311	.037	.092	.239	.050
**Pallidum**	**total**	.307	**.372** **	.117	−.008	.025	.242	−.267	.403	.246	.083	−.304	.130
	**left**	**.346** *	**.394** **	.073	−.125	.048	.233	−.264	.228	.253	.058	−.299	−.003
	**right**	.164	**.285** *	.152	.093	.001	.212	−.243	**.518** *	.202	.097	−.279	.234

Nominal significant Pearson’s correlation coefficients in bold,

*indicate p-values ≤0.05,

**p≤0.01.

Sex differences were significant for the corresponding z-transformed correlation coefficients of the relation between caudate volumes (total, left and right) and fluid figural intelligence scores (p = 0.01) and left pallidum volumes and crystallised figural intelligence scores (p = 0.05).

**Figure 1:**
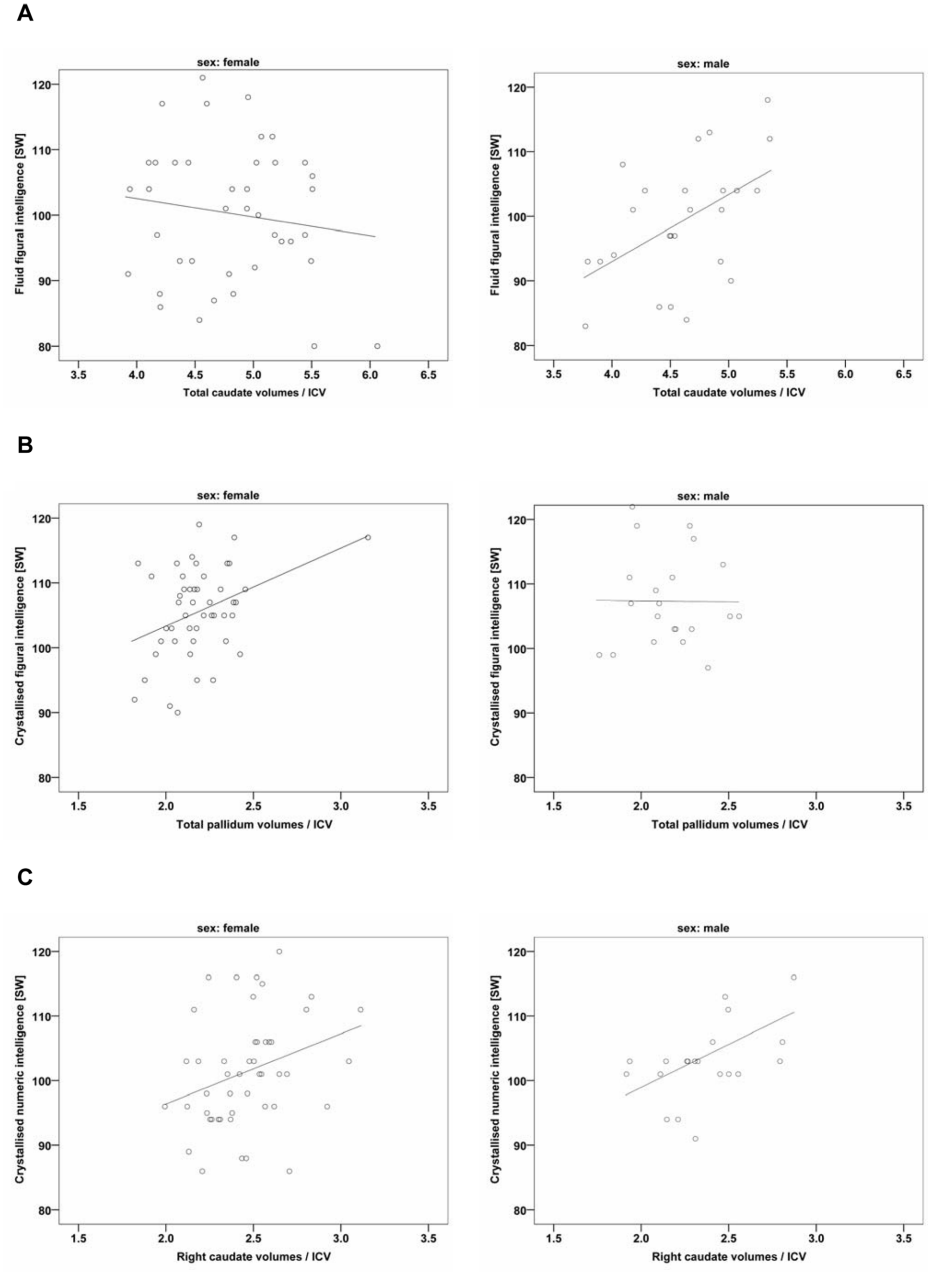
Correlations between basal ganglia volumes and different aspects of intelligence independent from prefrontal cortical influence. Scatterplots indicate the distribution of subcortical volumes and intelligence scores. **A.** Significant correlation between total caudate volumes and fluid figural intelligence scores only in males. **B.** Significant correlation between total pallidum volumes and crystallised figural intelligence only in females. **C.** Significant correlation between right caudate volumes and crystallised numeric intelligence in both males and females. ICV, intracranial volumes; SW, standard values (population mean = 100, SD = 10).

To account for a potential influence of the medial prefrontal cortex [28], i. e. medialorbitofrontal, lateralorbitofrontal and superiorfrontal cortical subfields, on the relation of intelligence performance and basal ganglia volumes, partial correlations were calculated. Basal ganglia volumes were highly correlated with medial prefrontal cortical volumes (Table S1). Partial correlations controlling for medial prefrontal cortical volumes increased the strength of the relations between caudate volumes and fluid figural intelligence in males and between pallidum volumes and crystallised figural intelligence scores in females. In contrast, the strength of the correlations between putamen volumes and crystallised intelligence in females declined and did not reach significant values (Table 3). Taken together, figural intelligence aspects appeared to be affected by sex and were associated with a specific combination of basal ganglia nuclei and intelligence factors (Figure 1A, 1B).

**Table 3:** Partial correlations between the basal ganglia nuclei volumes and different aspects of intelligence controlling for medial prefrontal cortical volumes.

		Figural intelligence		Numeric intelligence	
		female	male	Female	male
		crystallised	fluid	crystallised	crystallised
**Region**					
**Caudate**	**total**	.095	**.580** **	**.325** *	.497
	**left**	.018	**.615** **	.261	.416
	**right**	.160	**.525** *	**.361** *	**.524** *
**Putamen**	**total**	.272	.361	.210	.325
	**left**	.288	.415	.199	.366
	**right**	.245	.288	.213	.273
**Pallidum**	**total**	**.399** **	.081	.211	.285
	**left**	**.416** **	.088	.174	.087
	**right**	**.323** *	.068	.217	.443

Significant Pearson’s partial correlation coefficients in bold,

*indicate p-values ≤0.05,

**p≤0.01.

### Numeric but not Verbal Intelligence is Related to Basal Ganglia Volumes

We also found evidence for an association between basal ganglia volumes and numeric intelligence in our analysis. We revealed nominally significant correlations of the total caudate volumes with numeric intelligence for both females and males, but only for crystallised intelligence. Analysing lateralised volumes showed that this relationship was mostly driven by volumes of the right caudate, but correlations of the left caudate volumes showed a similar trend (Table 2). In males, there was an additional significant effect in the right pallidum. Significant correlations (p≤0.05; uncorrected) retained significance after FDR correction at q = 0.2 (critical p = 0.08).

Partial correlations controlling for medial prefrontal cortical volumes (Table S1) increased the strength of the relations in females; in males, significance was retained only for right caudate volumes (Table 3). Thus, the correlation between crystallised numeric intelligence and caudate volumes appeared to be right-lateralised (Figure 1C). In contrast, no associations with basal ganglia volumes were found for the verbal intelligence scores (Table 2).

### Validation of Study Results

In addition to the basal ganglia volumes, the hippocampal and prefrontal cortical volumes of our study participants were calculated for reasons of validation. The total hippocampal volumes and total scores for crystallised, but not fluid intelligence, were significantly correlated (r = 0.240, p = 0.05). This result was consistent with previous reports, which identified an involvement of the hippocampus in long-term memory and crystallised intelligence [29]. Fluid intelligence was reported to be associated with prefrontal cortex subfield volume [28], [30], which was replicated by our data. The total medialorbitofrontal cortical volumes correlated positively with the total scores for fluid intelligence (r = 0.276, p = 0.03).

## Discussion

In our exploratory study, we have found evidence for a close association between volumes of the caudate nucleus, putamen and pallidum, which are important subcortical nuclei of the basal ganglia system, and intelligence scores in young healthy individuals. This is an important finding because brain correlates of intelligence were mostly found in the cortex, with the frontal grey matter being the predominant region for executive and cognitive functions. Thus, our study highlights the importance of subcortical brain structures for cognitive abilities related to successful life coping. One strength of our study design is that our analysis of intelligence was based on the intelligence model of Cattell; Cattell developed a two-split model of intelligence, consisting of information processing speed (which is known as the fluid factor of intelligence) and accumulated knowledge (which is referred to as the crystallised factor of intelligence [17]) rather than measuring the general intelligence factor [31]. The sub-classification of both fluid and crystallised intelligence into verbal, numeric and figural intelligence aspects allowed for an even more differentiated approach. This intelligence analysis offered deep insights into the aspects of cognitive processing related to the basal ganglia nuclei.

The most intriguing result of the present analysis was that figural intelligence was significantly related to basal ganglia volumes in a sex-specific way. We observed significant correlations between fluid figural intelligence and the caudate, as well as crystallised figural intelligence and the putamen and pallidum. Thus, our data were consistent with the differentiated functions reported for the caudate nucleus and putamen. Whereas the caudate was shown to be involved in more executive functions and is highly connected to the prefrontal cortex, the putamen mediates learning processes [12]. However, this association varied across the two sexes: the putamen and pallidum volumes were significantly correlated with the crystallised figural aspects of intelligence, but only in females. The caudate volumes showed an association with fluid figural intelligence scores, but only in males. This result is further supported by a study describing an association between caudate volumes and performance in a spatial navigation task [32].

For the numeric aspects of intelligence, no differences between females and males regarding their relations of volumetric and intelligence data were detected in this analysis. In both sexes, a close association between the caudate volumes and crystallised numeric intelligence was shown, which was pronounced for the right caudate. Parietal cortical regions activated during verbal coding of numbers are shown to be left-lateralised [33]. This gives a hint that subcortical regions might play a role mainly for non-verbal number processing with the right caudate nucleus being important for general numeric performance.

Verbal aspects of our analysis were not correlated with basal ganglia volumes. This finding was supported by a recent anatomical and functional imaging study conducted by Moore *et al.* [34], who observed that during verbal memory tasks, the basal ganglia were involved in encoding processes, but not in the retrieval of verbal contents. The results are also in line with earlier studies, which did not indicate a relation between caudate volumes and verbal IQ scores [2].Taken together, verbal intelligence may be associated exclusively with cortical brain regions, in contrast to the figural and numeric aspects of intelligence, which were shown in this study to be influenced by subcortical brain structures.

Since the prefrontal cortex is thought to be the main region of cognition, we included partial correlation analyses to control for its influence on the relation between intelligence and basal ganglia. Volumes of the medial prefrontal cortex were significantly correlated with basal ganglia volumes and total fluid intelligence scores. Nevertheless, our data revealed a strong relation between basal ganglia and certain aspects of intelligence even when controlling for this prefrontal effect. Thus, the medial prefrontal cortex did not mediate the relation between figural intelligence and caudate nucleus (male) and pallidum (female), but seemed to exert some influence on the relation with putamen volumes (female). The relation between crystallised numeric intelligence with right caudate volumes appeared to be independent from prefrontal influences, but the effect of total caudate volumes in males was partially mediated by the prefrontal cortex. This stresses the significant impact of selected subcortical brain structures on specific cognitive performance.

Our study is the first report to explore the potential correlation between basal ganglia nuclei and the described differentiated intelligence assessments with a focus on sex-specific aspects. However, associations between sex and intelligence have always been a highly discussed topic [35], [36]. But in contrast to what the stereotypes about gender and intelligence claim, only few aspects regarding sex-specific intelligence differences were confirmed; in particular, males perform better in typical spatial tasks [37], [38] and females in verbal tasks [25], [39]. There is growing evidence that neural correlates of general intelligence differ between the sexes [40] and that females code information in a more efficient way, requiring less neural substance for achieving similar output [41]. Thus, females and males may – as seen in our results regarding figural tasks – encode particularly spatial information in a different manner. A potential explanation for this difference may be that specific cognitive performance relies on sex-specific encoding and learning processes in specific brain regions that influence the volume of these nuclei. Spatial and figural performance is also influenced by environmental factors, as special training can influence spatial cognition and revert sex-specific differences [42]. But the causality could also be reversed, indicating a basal distinct effect of sex-specific gene expression, which guides brain development and subsequently cognitive encoding processes. This raises the question of mediating endophenotypes. Genes affecting brain structures were extensively studied within the last several years. Several candidate genes that influence brain volumes have been identified [43]. Also the basal ganglia volumes are thought to be influenced by a variety of genes and could mediate potential genetic and sex-specific effects on cognitive performance.

Our study results were validated by a close correlation of hippocampal volumes and the total score of crystallised intelligence, and of medial prefrontal cortical volumes and the total scores of fluid intelligence, as previously reported [28]–[30], [44]. This was consistent with the overall idea of crystallised intelligence as the ability to access stored memory. Learning and memory processes occur in the hippocampus, where memory contents are transformed from working memory into long-term memory. Crystallised intelligence is defined by the accumulated knowledge derived from long-term and stable memory contents. Growing evidence supports brain correlates of fluid intelligence mainly within the prefrontal cortex [28]. These findings emphasised that fluid and crystallised intelligence are distinct cognitive qualities with differential brain correlates [45]. Whereas fluid intelligence declines with age, crystallised intelligence increases during life with the accumulation of solid knowledge [46]. Because of this dependency on age regarding the performance in both intelligence factors, it was important to minimise the range of age. Intelligence influences success at school and education and vice versa. Thus, we selected medical students for our study, who were highly similar in their academic background. In total, the conclusions drawn from this study are also valid due to the homogenous study sample.

However, there were some limitations of our study that should be considered. We conducted an exploratory study, which showed several effects with nominal significance. It is an often-discussed topic which statistical threshold to apply on initial exploratory studies with multiple comparisons. A very strict threshold prevents from further investigating promising results, whereas a very permissive threshold increases the amount of false positive results [47]. It was thus suggested to apply a stepwise procedure on studies with multiple comparisons [48]. To keep the number of false negatives low at the first stage of exploratory studies, a correction for multiple testing using FDR with a moderate q-value may be applied [27]. Yet, the threshold should not exceed q = 0.2 [48]. FDR correction at q = 0.2 was applied in several exploratory studies on different topics with promising results [48]–[51]. In a second step, significant results at q = 0.2 can be tested and replicated in a follow-up study including the conservative Bonferroni correction for multiple comparisons [27]. Applying a moderate FDR correction, our study points to interesting correlations between basal ganglia volumes and indicated intelligence aspects. These correlations should be further investigated in an independent follow-up study. In addition, the study could be expanded using functional imaging, additional cognitive assessments and genetic analyses to gain deeper insight into the relationship between the basal ganglia and intelligence.

Our results may also be relevant for clinical aspects. In autism, the volume of the caudate nucleus is increased as shown in various studies [16], [24], [52]–[54]. It may potentially not be an adverse aspect of the disease, but it is consistent with the observation that autistic individuals often show giftedness in the field of mathematics or spatial tasks. In addition, verbal aspects are mostly underdeveloped, which is consistent with the lack of a relationship between basal ganglia volumes and verbal performance. Interestingly, in Parkinson’s disease, visual learning was reported to be impaired [15]. In particular, for figural intelligence, the visual component of perception is very important for a good cognitive performance. This could serve as indirect evidence for the importance of an intact basal ganglia system for figural intelligence.

Taken together, our study provides the first evidence for an association between basal ganglia nuclei and essential cognitive abilities in the field of figural and numeric performance. The sex-specific effects observed in this study further provide insight on the mosaic of sex and intelligence.

## Supporting Information

Table S1 **Correlations between basal ganglia nuclei volumes and medial prefrontal cortical subfield volumes.**
(DOCX)
