# Supplementary material for: Neuroanatomical Correlates of Intelligence in Healthy Young Adults: The Role of Basal Ganglia Volume
Source: PLoS One. 2014 Apr 3;9(4):e93623. doi: 10.1371/journal.pone.0093623 (PMC3974758; doi:10.1371/journal.pone.0093623)
Supplement: Table S1 — Correlations between basal ganglia nuclei volumes and medial prefrontal cortical subfield volumes. (DOCX) [file pone.0093623.s001.docx]

**Supplementary Tables**

**Table S1. Correlations between basal ganglia nuclei volumes and medial prefrontal cortical subfield volumes.**

|  | **Medialorbitofrontal PFC** | | **Lateralorbitofrontal PFC** | | **Superiorfrontal PFC** | |
| --- | --- | --- | --- | --- | --- | --- |
|  | **female** | **male** | **female** | **male** | **female** | **male** |
| **Caudate** | **.306 **** | **.476 **** | **.361 **** | **.525 **** | **.416 **** | **.513 **** |
| **Putamen** | **.362 **** | **.446 **** | **.349 **** | **.346 *** | **.436 **** | **.445 **** |
| **Pallidum** | **.256 *** | **.421 **** | **.338 **** | **.345 *** | **.341 **** | **.382 **** |

Significant Pearson’s correlation coefficients in bold, * indicate p-values ≤ 0.05, ** p ≤ 0.01. PFC, prefrontal cortex. FDR correction for multiple comparisons at q = 0.05 was applied.
